# Colocalization and Disposition of Cellulosomes in *Clostridium clariflavum* as Revealed by Correlative Superresolution Imaging

**DOI:** 10.1128/mBio.00012-18

**Published:** 2018-02-06

**Authors:** Lior Artzi, Tali Dadosh, Elad Milrot, Sarah Moraïs, Smadar Levin-Zaidman, Ely Morag, Edward A. Bayer

**Affiliations:** a Department of Biomolecular Sciences, The Weizmann Institute of Science, Rehovot, Israel; b Department of Chemical Research Support, The Weizmann Institute of Science, Rehovot, Israel; University of Delaware

**Keywords:** CLEM, SEM, STORM, bacterial cell surface, fluorescence microscopy, protuberance-like structures

## Abstract

Cellulosomes are multienzyme complexes produced by anaerobic, cellulolytic bacteria for highly efficient breakdown of plant cell wall polysaccharides. *Clostridium clariflavum* is an anaerobic, thermophilic bacterium that produces the largest assembled cellulosome complex in nature to date, comprising three types of scaffoldins: a primary scaffoldin, ScaA; an adaptor scaffoldin, ScaB; and a cell surface anchoring scaffoldin, ScaC. This complex can contain 160 polysaccharide-degrading enzymes. In previous studies, we proposed potential types of cellulosome assemblies in *C. clariflavum* and demonstrated that these complexes are released into the extracellular medium. In the present study, we explored the disposition of the highly structured, four-tiered cell-anchored cellulosome complex of this bacterium. Four separate, integral cellulosome components were subjected to immunolabeling: ScaA, ScaB, ScaC, and the cellulosome’s most prominent enzyme, GH48. Imaging of the cells by correlating scanning electron microscopy and three-dimensional (3D) superresolution fluorescence microscopy revealed that some of the protuberance-like structures on the cell surface represent cellulosomes and that the components are highly colocalized and organized by a defined hierarchy on the cell surface. The display of the cellulosome on the cell surface was found to differ between cells grown on soluble or insoluble substrates. Cell growth on microcrystalline cellulose and wheat straw exhibited dramatic enhancement in the amount of cellulosomes displayed on the bacterial cell surface.

## IMPORTANCE

Conversion of plant biomass into soluble sugars is of high interest for production of fermentable industrial materials, such as biofuels. Biofuels are a very attractive alternative to fossil fuels, both for recycling of agricultural wastes and as a source of sustainable energy. Cellulosomes are among the most efficient enzymatic degraders of biomass known to date, due to the incorporation of a multiplicity of enzymes into a potent, multifunctional nanomachine. The intimate association with the bacterial cell surface is inherent in its efficient action on lignocellulosic substrates, although this property has not been properly addressed experimentally. The dramatic increase in cellulosome performance on recalcitrant feedstocks is critical for the design of cost-effective processes for efficient biomass degradation.

## INTRODUCTION

Cellulosomes are highly structured protein complexes produced by specialized anaerobic bacteria to efficiently break down plant cell wall polysaccharides. These multienzyme assemblies are composed of an enormous collection of enzymatic subunits that are joined together by nonenzymatic structural subunits, called “scaffoldins” (1–3). Assembly of these components relies on the existence of two complementary modules—cohesins in the scaffoldin and a dockerin in each cellulosomal enzyme—that interact strongly and specifically (4–6). Cellulosomes become increasingly elaborate when additional scaffoldins are involved in complexation, which amplifies the number of enzymes in a single complex.

*Clostridium clariflavum* and *Clostridium thermocellum* are the two known thermophilic anaerobic cellulosome-producing bacterial species that have been studied for potential industrial application (7–11). *C. clariflavum* produces a variety of diverse cellulosome complexes, some of which are presumably anchored to the bacterial cell surface, whereas others appear to be intrinsically cell free (12, 13).

**FIG 1:**
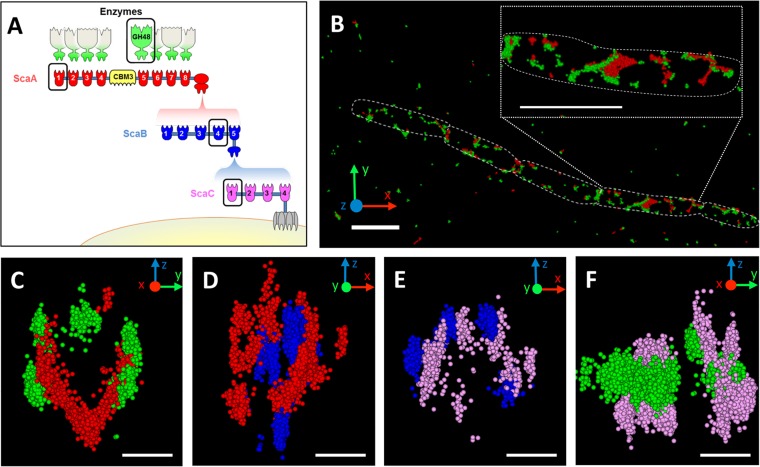
Hierarchical organization of cell surface cellulosome components. (A) Schematic illustration of the major cellulosome system of *C. clariflavum*. Components circled in black were immunolabeled during experiments. Cellulosome components are color coded to match the labeling pattern in subsequent panels. (B) Representative 3D STORM image of cellobiose-grown cells labeled with anti-GH48 (green) and anti-CohA (red). Scale bar, 2 µm. (C to F) Representative STORM cross-sectioned images of selected bacterial cells immunolabeled with pairs of antibodies (scale bars, 300 µm). A portion of each bacterium was sectioned and rotated 90°, resulting in a view through its long axis, as demonstrated for the GH48/CohA-labeled bacterium in Movie S1. (C) Anti-GH48 (green) and anti-CohA (red); (D) anti-CohA (red) and anti-CohB (blue); (E) anti-CohB (blue) and anti-CohC (pink); (F) anti-CohC (pink) and anti-GH48 (green). CohA and CohC were labeled with primary monoclonal mouse antibodies and Alexa 568-conjugated anti-mouse secondary antibodies. CohB and GH48 were labeled with polyclonal chicken antibodies and Alexa 647-conjugated anti-chicken secondary antibodies. See Movies S1 to S4 for various viewing perspectives of the labeled cells shown in panels C to F, respectively.

The major cell-attached cellulosome complex of *C. clariflavum* is composed of 185 proteins when fully occupied. This particularly elaborate complex comprises interconnecting scaffoldins, which include a single cell-anchored ScaC, 4 ScaBs (adaptor scaffoldins), 20 ScaAs (primary scaffoldins), and 160 enzymatic subunits that interact with the eight ScaA cohesins. The proposed assembly of the complex on the cell surface is displayed in Fig. 1A.

10.1128/mBio.00012-18.6 MOVIE S1  3D STORM representation of cellobiose-grown cells labeled with anti-GH48 (green) and anti-CohA (red). The movie shows the rotation of the bacterium along its long axis. A short segment of the bacterium was then selected and orientated such that the view is directed through the long axis of the bacterium. This viewing perspective shows the hierarchy of the designated components on the cell surface (Fig. 1C). Download MOVIE S1, MOV file, 0.2 MB. Copyright © 2018 Artzi et al. 2018 Artzi et al. This content is distributed under the terms of the Creative Commons Attribution 4.0 International license.

10.1128/mBio.00012-18.7 MOVIE S2  3D STORM representation of cellobiose-grown cells labeled with anti-CohA (red) and anti-CohB (blue). Different viewing perspectives of the 3D STORM image are presented by rotating and zooming in on the image (Fig. 1D). Download MOVIE S2, MOV file, 0.1 MB. Copyright © 2018 Artzi et al. 2018 Artzi et al. This content is distributed under the terms of the Creative Commons Attribution 4.0 International license.

10.1128/mBio.00012-18.8 MOVIE S3  3D STORM representation of cellobiose-grown cells labeled with anti-CohB (blue) and anti-CohC (pink). Different viewing perspectives of the 3D STORM image are presented by rotating the image (Fig. 1E). Download MOVIE S3, MOV file, 0.8 MB. Copyright © 2018 Artzi et al. 2018 Artzi et al. This content is distributed under the terms of the Creative Commons Attribution 4.0 International license.

10.1128/mBio.00012-18.9 MOVIE S4  3D STORM representation of cellobiose-grown cells labeled with anti-CohC (pink) and anti-GH48 (green). Different viewing perspectives of the 3D STORM image are presented by rotating the image (Fig. 1F). Download MOVIE S4, MOV file, 0.3 MB. Copyright © 2018 Artzi et al. 2018 Artzi et al. This content is distributed under the terms of the Creative Commons Attribution 4.0 International license.

The *C. clariflavum* cellulosome represents the largest and most intricate discovered to date. Tremendous efforts have been dedicated over the years to characterize cellulosomes that are released from the cell surface, i.e., cell-free complexes (14–17). However, cell-attached cellulosomes essentially remain a mystery. Previously, surface-attached cellulosomes were imaged by scanning electron microscopy (SEM) and transmission electron microscopy, usually without specific labeling or by immunolabeling using a single “cellulosome-specific” antibody (18–24). In this work, we examined the display of cellulosome complexes on the *C. clariflavum* cell surface and observed the structural organization and colocalization of the cellulosome proteins by using a set of antibodies elicited against separate interacting cellulosomal components.

Study of the colocalization of two proteins has been challenging, due to the diffraction limits of resolution of the standard fluorescent and confocal microscopes (~300 nm) (25). Consequently, two closely colocalized proteins would be viewed as a single point, comprising the mixed fluorophores (e.g., combined red and green fluorophores will appear as a single yellow spot). To overcome these drawbacks, we applied superresolution microscopy (i.e., STORM [stochastic optical reconstruction microscopy]), which generates ~20-nm resolution and allows more accurate and elaborate observations of colocalized macromolecules (26).

*C. clariflavum* was also imaged by scanning electron microscopy (SEM), which revealed morphological protuberances on the bacterial cell surface, consistent with similar structures observed previously in other cellulosome-producing bacteria (18–21). In order to explore whether these protuberances are cellulosomes, we developed a standardized protocol for correlative light and electron microscopy (CLEM) of STORM and SEM (27–29). This approach enabled us to correlate between the specific labeling of cellulosome components by STORM and the observed protuberances on the bacterial cell surface by SEM. CLEM takes advantage of two complementary microscopy methods to produce a more extensive picture of a biological specimen via precise fluorescence labeling of target proteins and very-high-resolution morphological imaging of the specimen. Combining the two methodologies allowed us to identify the presence of cellulosomes on some but not all of the *C. clariflavum* cell surface protuberances.

The goal of this study was to demonstrate the abundance and distribution of cellulosomes attached to the cell surface of *C. clariflavum* and to examine whether the complex is composed of the scaffoldin and enzyme components at the expected hierarchy of assembly that was determined earlier by bioinformatic, biochemical, and proteomic analyses (12, 13). In previous studies, cellulosome composition was demonstrated to be altered upon cultivation of the bacterium on different carbon sources (30, 31). We therefore examined how bacterial growth on simple versus recalcitrant, insoluble substrates affected display of the cellulosomes on the cell surface.

## RESULTS AND DISCUSSION

### Selection of cellulosomal components for labeling.

We selected 4 proteins critical for the assembly and function of the major cellulosome complex of *C. clariflavum*: ScaA (Clocl_3306), ScaB (Clocl_3305), ScaC (Clocl_3304), and the most abundant enzyme in the cellulosome, GH48 (Clocl_4007). A specific module of each of the latter scaffoldins was cloned and expressed as antigen for antibody production: the first cohesin of ScaA (CohA), the fourth cohesin of ScaB (CohB), and the first cohesin of ScaC (CohC). Mouse monoclonal antibodies were produced against CohA and CohC, and chicken polyclonal antibodies were produced against CohB and GH48. Antibody specificity was tested by enzyme-linked immunosorbent assay (ELISA), whereby each of the antigens was incubated with all produced antibodies to check for cross-reactivity. All antibodies exhibited high specificity only to their matching antigen (see Fig. S1 in the supplemental material). The diversity in antibody origin allowed us to label the bacterial cell surface with pairs of antibodies and to compare their location on the cell surface, their presumed colocalization, and their relative disposition (i.e., proximity to the cell surface). The cells were immunolabeled with the (i) anti-GH48 and anti-CohA, (ii) anti-CohA and anti-CohB, (iii) anti-CohB and anti-CohC, and (iv) anti-GH48 and anti-CohC pairs and imaged by STORM. In addition to the capacity for three-dimensional (3D) imaging, the STORM technique is ideal for protein colocalization studies of small entities, due to its exceptionally high resolution compared to conventional fluorescence microscopy (25). Preparation of samples for imaging was challenging, since the use of fixatives resulted in failure of the antibodies to bind the target antigens. Therefore, experiments were performed on cells in their native form that were fixed only subsequently to immunolabeling.

10.1128/mBio.00012-18.2 FIG S1  Antibody specificity. The two monoclonal mouse antibodies (anti-CohA and anti-CohC) and the two polyclonal chicken antibodies (anti-CohB and anti-GH48) were tested for possible cross-reactivity against all four antigens: CohA (A), CohB (B), CohC (C), and GH48 (D). No cross-reactivity was detected, and each antibody bound specifically only to its matching antigen. Error bars present standard deviations from three biological experiments in triplicate. Download FIG S1, PDF file, 0.2 MB. Copyright © 2018 Artzi et al. 2018 Artzi et al. This content is distributed under the terms of the Creative Commons Attribution 4.0 International license.

### Organization of cellulosome proteins on the cell surface.

For each dual-immunofluorescent labeling experiment, the location of one component relative to the other was compared. According to the assembly model of the *C. clariflavum* cellulosome, ScaC is expected to be closest to the bacterial cell surface, since it anchors the complex to the S-layer by the SLH module. ScaB is located distal to ScaC, followed by ScaA and the enzymes at the most exterior layer on the cell surface (Fig. 1A). A representative STORM image of a *C. clariflavum* cell grown on cellobiose, in which GH48 and CohA are immunolabeled with the corresponding antibodies, is presented in Fig. 1B. Most of the detected GH48 and CohA are colocalized.

To assess relative positioning of all 4 components, cross sections of cellobiose-grown bacteria labeled with corresponding pairs of antibodies are presented in Fig. 1C to F. In Fig. 1C, the GH48/CohA pair is immunolabeled, and it is clearly observed that ScaA surrounds the bacterial cell and is generally enveloped by GH48. Furthermore, when the CohA/CohB pair is labeled (Fig. 1D), ScaB is closer to the cell surface and is surrounded by ScaA. It can thus be concluded from these two images that ScaA is located between GH48 and ScaB. Continuing with the CohB/CohC pair (Fig. 1E), ScaC appears proximal to the cell relative to ScaB. Thus, ScaB is located between ScaC and ScaA. Labeling the CohC/GH48 pair (Fig. 1F) reinforces the latter observations that ScaC is clearly interior compared to the GH48 enzyme. ScaC thus functions as a platform for the other cellulosome components and anchors the complex to the cell surface. Movies S1 to S4 in the supplemental material provide different viewing perspectives of the four pairs of cellulosome components on the surface of cellobiose-grown cells. The results correspond precisely with the suggested model of *C. clariflavum* cellulosome assembly, which was based on cohesin-dockerin interaction studies (12, 13), and confirm the production of the major cell surface cellulosome, in the expected order of assembly, thus creating the largest cellulosome known in nature, with the ability to contain 160 enzymes.

### Cell surface cellulosome varies among cells grown on different substrates.

*C. clariflavum* is capable of growing on different carbon sources, which include cellulosic substrates and plant materials. It was shown previously that cellulosome-producing bacteria can adapt to grow on and utilize different carbon sources, due to their σ factors and the anti-σ (RsgI) sensory regulation system that senses the extracellular environment and activates production of required cellulosome proteins (32, 33). We showed that the composition of *C. clariflavum*-secreted cellulosomes varies when different carbon sources are supplemented for bacterial growth (13). In the current study, we were interested in exploring whether the display of cell surface cellulosomes changes as a function of the carbon source upon which the bacterium is grown. For this purpose, cells were immunolabeled with all four pairs of antibodies, and *C. clariflavum* was cultivated on three carbon sources: cellobiose, microcrystalline cellulose (MCC), and acid-pretreated wheat straw (WS).

In order to decide at which time point of the growth curve samples should be examined, we followed the enzymatic activity of cell-free and cell-attached cellulosomes as a function of bacterial cell growth (see Fig. S2 in the supplemental material). The logarithmic phase of cellobiose-grown cells takes place between 8 and 18 h, and cellulosome production seems to complement this time frame. We therefore selected the 12-h point for our analysis. Interestingly, comparison of catalytic (cellulase and xylanase) activity between the spent growth medium and the cell pellet as a function of time revealed that, in cellobiose-grown cells, most of the cellulosomes and enzymes are secreted and released into the medium. The cell pellet, however, demonstrated significantly lower ability to degrade xylan, and almost no catalytic activity was detected on MCC (Fig. S2).

10.1128/mBio.00012-18.3 FIG S2  Growth progress and cellulosome production of *C. clariflavum*. Cells were grown on cellobiose-containing medium, and growth progress was assessed by sampling the culture at different time points. (A) Absorbance (*A*_600_) at each time point. The graph shows the mean of each time point for three biological repeats. For each sample, the cells were removed from the supernatant fluids, and catalytic activity of both the supernatant and pellet was examined on microcrystalline cellulose (B) and xylan (C). The graphs in panels B and C are representative of three biological repeats. Standard deviations are presented. Download FIG S2, PDF file, 0.2 MB. Copyright © 2018 Artzi et al. 2018 Artzi et al. This content is distributed under the terms of the Creative Commons Attribution 4.0 International license.

Monitoring growth and enzymatic activity of cells grown on insoluble substrates is challenging, since it is not possible to measure the optical density of the culture. Measurements of total proteins (or enzymatic activity) of cells can be deceiving, since small MCC/WS particles precipitate with the cells and are covered with attached cell-free cellulosomes. DNA measurements are also problematic, since the WS DNA can interfere. While collecting cellulosomes for proteomic analysis, we monitored the growth process of *C. clariflavum* on MCC by measuring the time course of enzymatic activity of the spent growth media and by following the NaOH consumption during growth, which ended at ~50 h. For the present experiment, a time point of ~35 to 40 h was chosen. Cells at all growth stages (individual, filamentous, or dividing cells) were observed, thus indicating active growth preceding the stationary phase.

**FIG 2:**
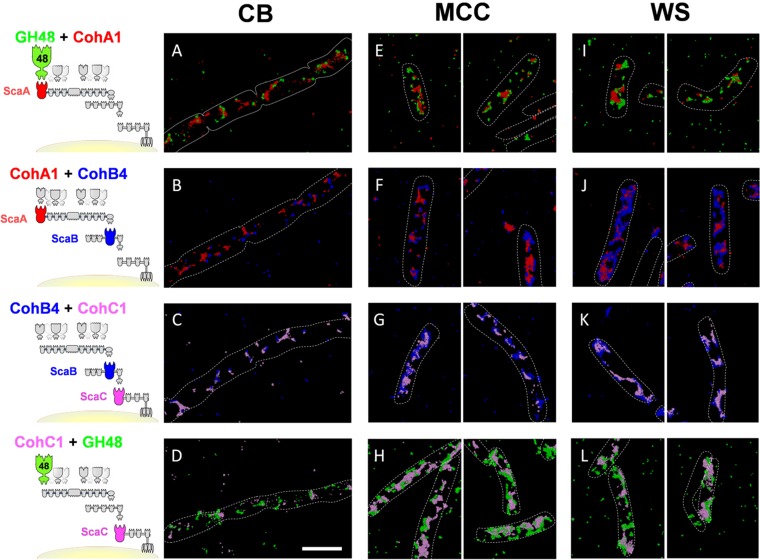
Cellulosome display on the bacterial cell surface as a function of the utilized carbon source. Shown are STORM 3D images of bacterial cells grown on cellobiose (CB [A to D]), MCC (E to H), and WS (I to L), labeled with four pairs of antibodies as indicated schematically. The images are representative of at least 2 biological repeats and two technical repeats. Scale bar, 2 µm. The target antigens are color coded according to the antibodies designated in the legend to Fig. 1 and the scheme shown on the left-hand side of this figure. See Movies S1 to S6 for various viewing perspectives of designated labeled cells grown on the different carbon sources.

Representative STORM images of immunolabeled cells grown on all substrates are presented in Fig. 2. Remarkable colocalization between each pair of antibodies is observed in cells grown on all three substrates. STORM images of cellobiose-grown cells (Fig. 2A to D) show clearly that cellulosome display on their cell surface occurs in significantly smaller amounts than in MCC- and WS-grown cells. Labeled cellulosome clusters appear localized and restricted and can be described as cellulosome “islands.” In contrast, MCC- and WS-grown cells (Fig. 2E to H and Fig. 2I to L, respectively) display much larger amounts of cell surface cellulosomes. The clusters are less local, more structured, and distributed across the cells. The differences between cells grown on soluble versus insoluble substrates are particularly emphasized upon comparison of the labeling profile of CohC: cellobiose-grown cells immunolabeled with anti-CohC display limited numbers of labeled sites (Fig. 2C and D), whereas MCC- and WS-grown cells show extensive labeling of the cell surface (Fig. 2G and H and Fig. 2K and L, respectively). See also Movies S5 and S6 in the supplemental material compared to Movies S4 and S3, respectively.

10.1128/mBio.00012-18.10 MOVIE S5  3D STORM representation of MCC-grown cells labeled with anti-CohC (pink) and anti-GH48 (green). Different viewing perspectives of the 3D STORM image are presented by rotating and zooming in on the image (Fig. 2H). Download MOVIE S5, MOV file, 1 MB. Copyright © 2018 Artzi et al. 2018 Artzi et al. This content is distributed under the terms of the Creative Commons Attribution 4.0 International license.

10.1128/mBio.00012-18.11 MOVIE S6  3D STORM representation of WS-grown cells labeled with anti-CohB (blue) and anti-CohC (pink). Different viewing perspectives of the 3D STORM image are presented by rotating and zooming in on the image (Fig. 2K). Download MOVIE S6, MOV file, 0.6 MB. Copyright © 2018 Artzi et al. 2018 Artzi et al. This content is distributed under the terms of the Creative Commons Attribution 4.0 International license.

**FIG 3:**
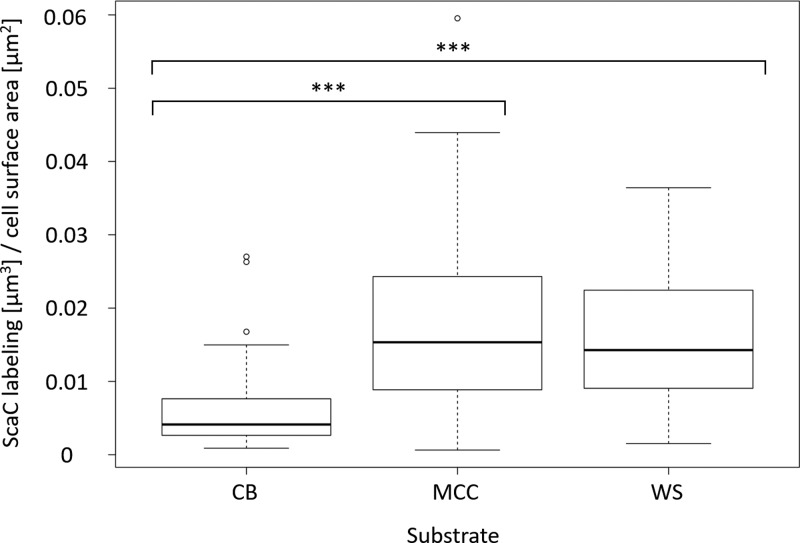
Display of ScaC on the *C. clariflavum* cell surface differs between cells grown on soluble or insoluble substrates. The coverage of cell surface area by ScaC was calculated for each bacterium by dividing the total volume of the detected ScaC clusters by the cell surface area of each bacterium. Groups of bacteria grown on the different carbon sources (cellobiose [CB], *n =* 94; MCC, *n =* 67; WS, *n =* 85; collected in at least 3 biological repeats) were compared by one-way analysis of variance (ANOVA), and Tukey’s multiple-comparison test was used to compare among all growth conditions. ***, *P* < 0.001. The total data set range (error bars) and the median value (horizontal black lines) are presented.

To measure quantitatively the extent of labeling on *C. clariflavum* cells grown on the different substrates, we calculated total ScaC coverage of each labeled bacterial cell surface (Fig. 3). ScaC was chosen for quantification, because as the anchoring scaffoldin responsible for attachment of the major cellulosome to the cell surface, ScaC would therefore represent the total amount of the surface-anchored complex. The coverage of cells grown on cellobiose by ScaC is indeed significantly lower than that of cells grown on MCC or WS. MCC- and WS-grown cells display similar levels of cell surface coverage by ScaC, indicating that when cells are grown on complex, insoluble substrates, they produce more cell-attached cellulosome complexes.

In order to ensure that cellulosome display patterns are not related to progress of bacterial growth, but to the carbon source *per se*, we also imaged cells grown on cellobiose for 18 h (late logarithmic/early stationary phase). At this time point, cellobiose-grown cells also displayed similar “islands” of cellulosomes, suggesting that the cell senses the carbon source in its environment and can regulate the required amounts of cell surface cellulosome. Reciprocally, we imaged cells grown on WS for only 24 h, which also displayed heavy cell surface cellulosome labeling at this early time point.

It is important to note that not all cells were labeled, and some were almost completely smooth. This can be due to the lack of fixation during immunolabeling, which may result in a leaching of cellulosomes from the cells during the washing steps. Indeed, in some of the samples we detected large dually labeled clusters remaining on the slide, and aggregates were found in the correlative SEM experiments that matched the labeled clusters. Alternatively, it is also possible that not all cells produce cellulosomes in the same manner and amounts. Bacterial populations that grow on the same carbon source may differ, where part of the cells produce cell-attached cellulosomes, whereas others can benefit secondarily from their cellulosomes, without the need to produce cell-attached cellulosomes of their own.

The observed differences in cellulosome display among cells grown on different substrates underscore the need for enhanced production of cell-attached cellulosomes by the bacterial cell as the substrate is more recalcitrant, insoluble, and complex. As demonstrated in Fig. S2, cells grown on cellobiose produce cellulosomes, but most of the cellulosomes are released into the growth medium, whereas the bacterial cell surface exhibits very low levels of enzymatic activity. As part of our previous study (13), we measured the catalytic activity in the growth medium at different time points during cell growth on cellobiose and MCC. Both cellobiose- and MCC-grown cells secrete similar amounts of cellulosomes into the extracellular growth medium, since similar amounts of released reducing sugars were detected. This information, together with our present results, leads us to conclude that cells may regulate the attachment of cellulosomes to the cell surface.

Accordingly when MCC- and WS-grown cells sense the various substrates via their array of membrane-embedded RsgI-specific carbohydrate-binding modules (CBMs) (32–34), cell-attached cellulosomes are produced in large quantities for intimate hydrolysis of the recalcitrant substrates and assimilation of the soluble sugar breakdown products for immediate use. Cells may therefore regulate cellulosome assembly and attachment to the cell surface as a function of sensing available carbon sources in the environment. To date, the σ-RsgI system is the only known regulatory system for cellulosome gene expression at the transcriptional level. It is currently unknown whether the cell has different levels of regulation for attachment of cellulosomes to the cell surface. ScaC, for example, is part of an operon with ScaA and ScaB, all of which are controlled under the same transcriptional regulation. Cellobiose-grown cells exhibit very limited display of ScaC on the cell surface. From proteomic analysis (13), ScaC is known to exist in very small quantities in the spent growth medium, in contrast to ScaA and ScaB, which are found in quantities similar to those of secreted cellulosomes from MCC-grown cells. This may suggest that the level of ScaC on the cell surface is regulated at the posttranscriptional (35), translational, or posttranslational level. Addressing these questions requires deeper understanding of the polysaccharide and oligosaccharide sensory machineries in *C. clariflavum* and further experimental exploration in the future.

### SEM imaging of bacteria grown on different carbon sources.

**FIG 4:**
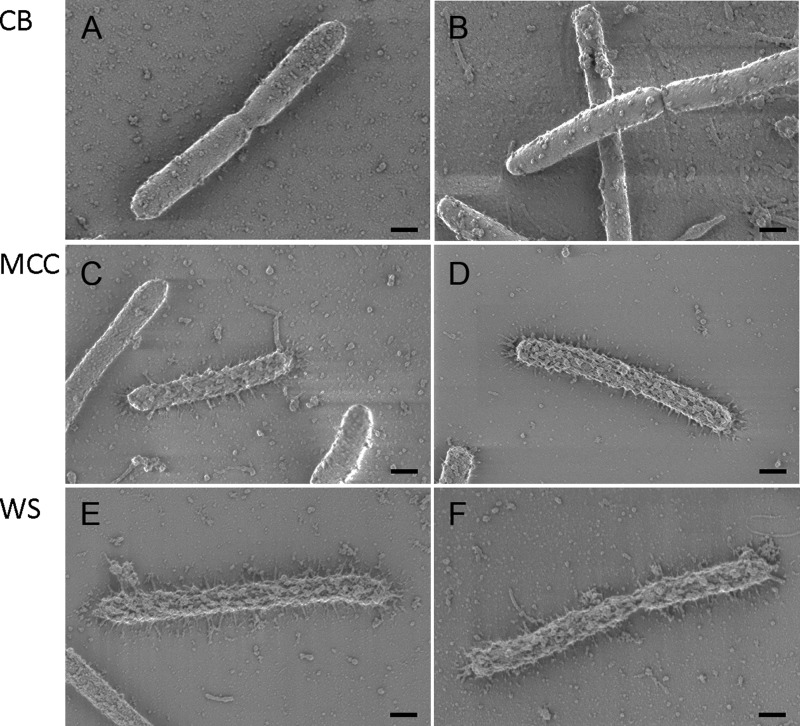
SEM imaging of cells cultivated on different carbon sources. Cells grown on cellobiose (CB [A and B]), MCC (C and D), and WS (E and F) were imaged by SEM. Representative images of at least two technical repeats are shown. Scale bars, 400 nm.

STORM images show accurate colocalization of the cellulosome particles, but do not provide information about the morphological properties of the bacterium and the display and distribution of cellulosome complexes that are independent of antibody labeling. For this reason, we imaged *C. clariflavum* by SEM to collect information about the general morphology and topography of the cells. We cultivated *C. clariflavum* on the same carbon sources used for STORM imaging (cellobiose, MCC, and WS), and the cells were prepared for SEM. Images of two representative cells grown on each of the substrates are presented in Fig. 4. The results of the SEM imaging correlated to the STORM imaging: cells grown on cellobiose (Fig. 4A and B) display protuberances on the cell surface that resembled, in their distribution and form, the immunolabeled clusters of cellulosome components in STORM—localized, relatively round, and distant from one another. Likewise, the SEM results for cells grown on insoluble complex carbohydrates resemble those of the STORM imaging. Thus, the distribution of protuberances is much more extensive on cells grown on MCC (Fig. 4C and D), where the cells are almost completely covered with protuberances. Cells grown on WS (Fig. 4E and F) show a similar coverage of protuberances on the cell surface, which appear even more crowded and developed than those of MCC-grown cells. In addition, not all imaged cells exhibited protuberances on their cell surfaces, which corresponds with the STORM imaging, where not all cells were immunolabeled. The lack of protuberances in negative-control *Escherichia coli* cells indicated that those observed on the *C. clariflavum* surface are not a result of sample preparation (see Fig. S3 in the supplemental material).

10.1128/mBio.00012-18.4 FIG S3  Scanning electron micrograph of *Escherichia coli* cells. *E. coli* cells comprised a negative control, prepared and imaged by SEM using a protocol identical to that of the *C. clariflavum* samples. The surface of the *E. coli* cell is smooth, thereby contradicting the possibility that the protuberances presented on *C. clariflavum* cells are artifacts of sample preparation. Scale bars, 2 µm. Download FIG S3, PDF file, 0.5 MB. Copyright © 2018 Artzi et al. 2018 Artzi et al. This content is distributed under the terms of the Creative Commons Attribution 4.0 International license.

### Correlation between STORM and SEM imaging.

In order to determine whether cell surface immunolabeling is situated together with the protuberances on specific bacterial cells and to demonstrate whether these protuberances represent specialized cellulosomes, we performed correlative light and electron microscopy (CLEM). The cells were first prepared and imaged by STORM, and the same samples were then processed for SEM imaging. The exact locations of each bacterium and the field that was imaged by STORM were determined by using grid-patterned coverslips.

**FIG 5:**
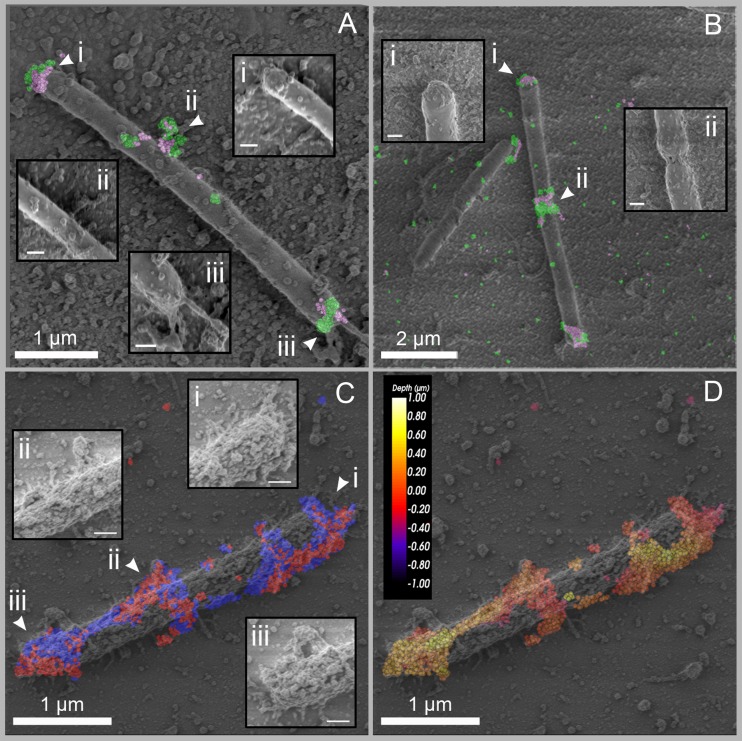
CLEM images of *C. clariflavum*. (A and B) Cellobiose-grown cells were immunolabeled with anti-GH48 (green) and anti-CohC (pink). (A) Scale bars, 1 µm (200 nm in each inset). (B) Scale bars, 2 µm (200 nm in each inset). (C and D) A WS-grown cell was immunolabeled with anti-CohA and anti-CohB. (C) Labeling is colored according to the specific probes: anti-CohA, red; anti-CohB, blue. (D) The depth of labeling is presented for the two combined probes, color coded from yellow (shallow labeling) to dark red (deep labeling, closer to the slide). Scale bars, 1 µm (200 nm in each inset in panel C).

In Fig. 5, two representative images revealed cells immunolabeled with the pair of anti-GH48 and anti-CohC antibodies, overlaid with their subsequent SEM images (Fig. 5A and B). The overlay was performed by aligning the bright-field light microscopy image with the SEM image. The fluorescent labeling demonstrates high colocalization of GH48 and CohC, whereas the SEM imaging shows small and local protuberances on the cell surface. Not all the protuberances are labeled by the cellulosome-specific antibodies. This observation cannot be explained by low antibody concentration, since MCC- and WS-grown cells are significantly more labeled than the cellobiose-grown cells, as demonstrated by their massive protuberances on the cell surfaces, labeled with the same antibody concentrations. One possible explanation would be that these protuberances represent other unrecognized protein complexes/aggregates. Alternatively, some represent unlabeled cellulosomes, possibly due to the steric arrangement of the enzymatic and scaffold components in the complex. Interestingly, in both Fig. 5A and B, the cellulosomes are particularly labeled at the cell poles and division sites. This observation was quite common among the imaged cellobiose-grown cells, but in addition, many cells exhibited cellulosomes scattered on the cell surface, as can be seen in Fig. 1B and Fig. 2A to D.

An example for CLEM imaging of a representative WS-grown cell is presented in Fig. 5C and D. In Fig. 5C, the cell is labeled with the anti-CohA/anti-CohB antibody pair. The cell is heavily labeled, and the labeling matches the morphological protuberances that are larger (Fig. 5C, insets) and more conspicuous than other protuberances on the cell surface. Three-dimensional STORM imaging also enables display of the labeling according to depth in the sample. In Fig. 5D, labeling depth of the two combined probes is shown. This view emphasizes the spatial localization of the complexes in the sample, whereby darker red-colored areas are closer to the glass surface, and lighter, yellow areas are situated upon the bacterial cell surface, further from the glass slide. The bacterium therefore displays cellulosomes around its cell surface, and not only on one plane. Some of the complexes are found closer to the slide surface and are labeled with a darker, pink color accordingly, as opposed to complexes that are detected on the cell surface and correlate with protuberances (labeled in yellow). Together with the SEM imaging, the depth representation of STORM contributes to the understanding of cellulosome organization around the cell. The STORM results highly correlate with the morphological information we get from the SEM imaging.

The imaging results achieved in this work revealed that the cellulosomes are frequently located at the cell poles (Fig. 5A and B). This observation is particularly apparent for cells grown on cellobiose. This phenomenon can be explained by the fact that most of the cellobiose-grown cells imaged are in the process of active growth and division. In this context, the cell probably produces new peptidoglycan in the center of the cell during growth, driving the old peptidoglycan toward the cell poles (36). Another explanation can be that cellulosome components are secreted via as-yet-unidentified transporters that are located at the cell poles, and they spread subsequently to the rest of the cell surface. In this context, protein secretion through a specific cell microdomain was discovered earlier in *Streptococcus pyogenes* by Rosch and Caparon (37) via the general secretory pathway (Sec pathway). A similar mechanism may serve to secrete cellulosomal components.

The results of this study provide definitive experimental proof of the presumed architecture of a cellulosome system, which had previously been based on the logical assessment of the experimentally determined cohesin-dockerin specificities of the component parts. Our results thus provide insight into the spatial organization of cellulosomes on the bacterial cell surface and reveal that the bacterial cell requires closely attached cellulosomes on its surface to break down highly recalcitrant substrates. This information is crucial for designing efficient plant cell wall deconstruction processes, such as consolidated bioprocessing, in which the bacterial cell, together with the secreted proteins, plays an important role in polysaccharide degradation.

## MATERIALS AND METHODS

### Cloning, expression, and purification of antigens.

Gene sequences of GH48, cohesin 1 of ScaA (CohA), cohesin 4 of ScaB (CohB), and cohesin 1 of ScaC (CohC) were cloned into a pET28 cassette. The purification of the four proteins was accomplished by using Ni-nitrilotriacetic acid (NTA) beads in a batch purification system as described earlier (38). Additional details for this and subsequent methods can be found in Text S1 in the supplemental material.

10.1128/mBio.00012-18.1 TEXT S1  Supplemental materials and methods. Download TEXT S1, PDF file, 0.1 MB. Copyright © 2018 Artzi et al. 2018 Artzi et al. This content is distributed under the terms of the Creative Commons Attribution 4.0 International license.

CohB and GH48 were used for the production of polyclonal chicken antibodies (Siap Laboratory, Bet Gamliel, Israel), while CohA and CohC were used for production of monoclonal mouse antibodies (Antibody Unit, Weizmann Institute).

### Cultivation of *C. clariflavum*.

*C. clariflavum* was cultivated on GS-2 medium as described previously (13), with three different carbon sources: 0.8% (wt/vol) cellobiose (MP Biomedicals, Illkirch, France), 0.1% microcrystalline cellulose (MCC) (Avicel, Sigma, Rehovot, Israel), or 0.5% acid-pretreated wheat straw. The chemical composition of hatched wheat straw (Valgaro, Poitiers, France) prior to acid pretreatment is described in reference 39. Acid pretreatment was performed with 5% (wt/vol) sulfuric acid as reported previously (13). Cells were grown at 55°C in 100 ml culture under anaerobic conditions for 12 h (on cellobiose) or 40 h (MCC and acid-pretreated wheat straw). Each medium was inoculated with 2% of culture (*A*_600_ of ~1.5 to 2).

### Fluorescence immunolabeling.

Briefly, cells were immunolabeled with pairs of antibodies: (i) anti-GH48 (1:500) plus anti-CohA (1:500), (ii) anti-CohA plus anti-CohB (1:500), (iii) anti-Coh-B4 plus anti-CohC (1:100), and (iv) anti-CohC plus anti-GH48. Cells were then postfixed with 3% paraformaldehyde (EMS, Hatfield, PA) and placed on coverslips attached to a petri dish, covered with 0.002% poly-l-lysine (P35G-1.5-14-C; MatTek *in vitro* Life Science Laboratories, Bratislava, Slovak Republic). For correlative work, grid-containing coverslips were used (81158; Ibidi, Martinsried, Germany), covered with poly-l-lysine. Detailed procedures can be found in Text S1 and Table S1 in the supplemental material.

10.1128/mBio.00012-18.5 TABLE S1  Primers used for cloning of the antigen genes. Download TABLE S1, PDF file, 0.1 MB. Copyright © 2018 Artzi et al. 2018 Artzi et al. This content is distributed under the terms of the Creative Commons Attribution 4.0 International license.

### Dual-color 3D STORM imaging and analysis.

Three-dimensional superresolution imaging was performed using a Vutara SR200 STORM microscope based on single-molecule localization biplane technology. Data were analyzed and visualized by the Vutara SRX software. Lateral localization accuracy was estimated by Thompson et al. (40) to be 5.94 ± 3.10 in our measurements.

### ScaC coverage calculation.

ScaC display on the cell surface was measured using the “Cluster analysis” module of the Vutara SRX statistical software. The “Cluster analysis” module is an “image-based” cluster identification algorithm designed to generate grouped clusters of localizations within the context of the larger data set. The clustering algorithm works by creating a binary image of the localization data by effectively binning the localization data. It then finds connected components in the binary image and works off those connected regions to correlate where the overall clusters within the data are located in the three-dimensional data set. The parameters used in the analysis are particle size (the size of the particles used when creating the density map) of 50 nm (30 nm for the cross section in Fig. 1), a minimum of 15 particle counts per cluster, opacity of 0.6, and an accumulation threshold (to distinguish clusters from background in the density map) of 0.08.

Each ScaC cluster was identified, and its volume was calculated. The total volume of ScaC clusters per single bacterium was divided by the cell surface area of a single bacterium using the equation ScaC coverage = [total cluster volume (μm^3^)]/[2π*r*⋅*l* (μm^2^) + 4π*r*^2^ (μm^2^)], where *r* is the radius and *l* is the length of the bacterium.

Statistical analysis of log ScaC coverage was performed by the R program version 3.2.4: by one-way analysis of variance (ANOVA), *P* < 0.001; by Tukey’s multiple-comparison test, for MCC-cellobiose and WS-cellobiose, *P* < 0.001, and for WS-MCC, *P* = 0.995.

### Correlative STORM and SEM imaging.

Bacteria were adhered to grid-containing coverslips (81158; Ibidi) covered with 0.002% poly-l-lysine. After STORM imaging (as described above), the samples were fixed with a solution containing 2% glutaraldehyde (EMS) and 4% paraformaldehyde in 0.1 M cacodylate buffer for 4 days. Samples were washed in double-distilled water three times to remove fixative solution. Coverslips were separated from the petri dishes using glass bottom fluid (MatTek DCF-OS-30), and dehydration of the samples was carried out in increasing ethanol concentrations (30, 50, 70, 90, 95, and 100% [vol/vol]), followed by critical point drying using CPD30 (Bal-Tec). Samples were sputter-coated with 5 nm Cr and visualized in the high-resolution Gemini SEM 500 (Carl Zeiss, Inc., Microscopy GmbH, Oberkochen, Germany). Imaging was carried out using an SE In-Lens detector at an accelerating voltage of 0.3 kV.

Cells, from which the STORM data were collected, were located in the SEM, using the grid markers on the glass slides. Overlay of the data was performed by first matching the bright-field image of the bacteria with the SEM image, followed by superimposing the STORM image. The correlation between the topographical protuberances on the cell surface and the double-fluorescence labeling of the cellulosome complex was then assessed.
