# Supplementary material for: Colocalization and Disposition of Cellulosomes in Clostridium clariflavum as Revealed by Correlative Superresolution Imaging
Source: mBio. 2018 Feb 6;9(1):e00012-18. doi: 10.1128/mBio.00012-18 (PMC5801460; doi:10.1128/mBio.00012-18)
Supplement: FIG S1 [file mbo001183712sf1.pdf]

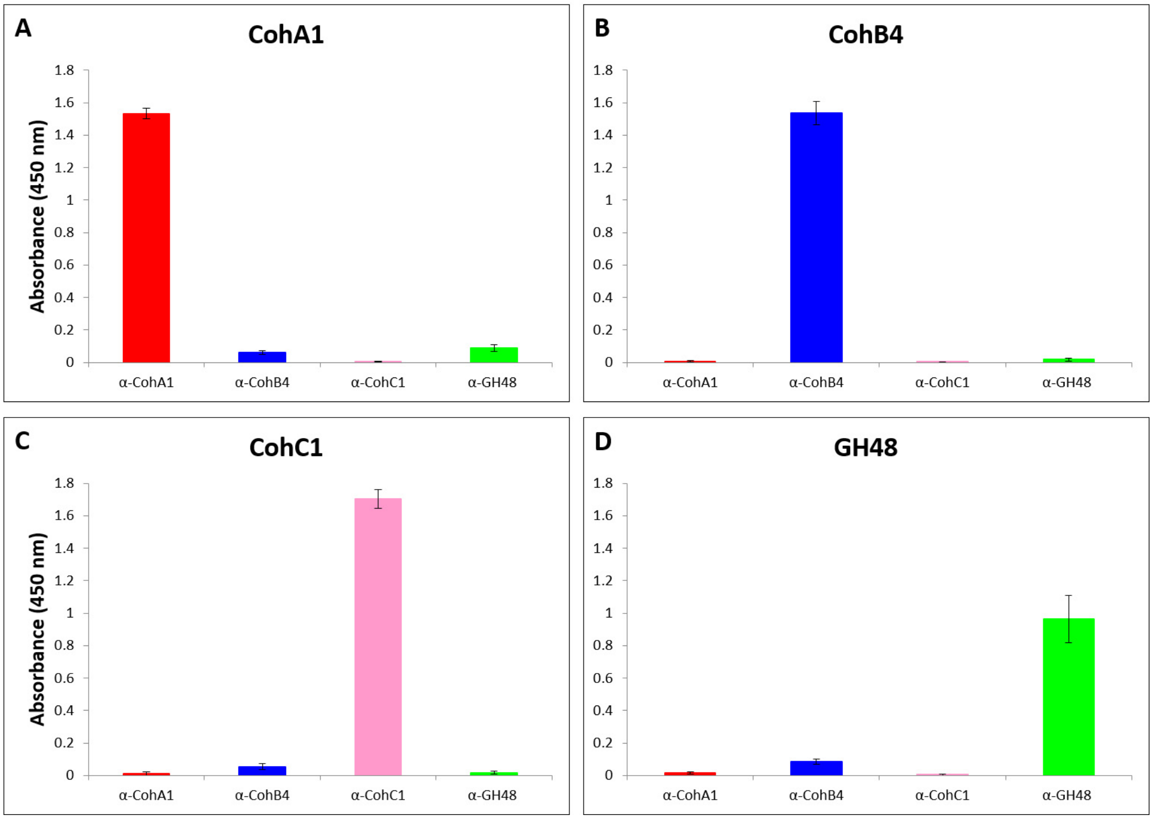

Figure S1. Antibody specificity. The two monoclonal mouse antibodies (anti-CohA and anti-CohC) and the two polyclonal chicken antibodies (anti-CohB and anti-GH48) were tested for possible cross-reactivity against all four antigens – CohA (A), CohB (B), CohC (C) and GH48 (D). No cross-reactivity was detected, and each antibody bound specifically only to its matching antigen. Error bars present standard deviations of three biological experiments in triplicate.
