# Supplementary material for: Colocalization and Disposition of Cellulosomes in Clostridium clariflavum as Revealed by Correlative Superresolution Imaging
Source: mBio. 2018 Feb 6;9(1):e00012-18. doi: 10.1128/mBio.00012-18 (PMC5801460; doi:10.1128/mBio.00012-18)
Supplement: FIG S2 [file mbo001183712sf2.pdf]

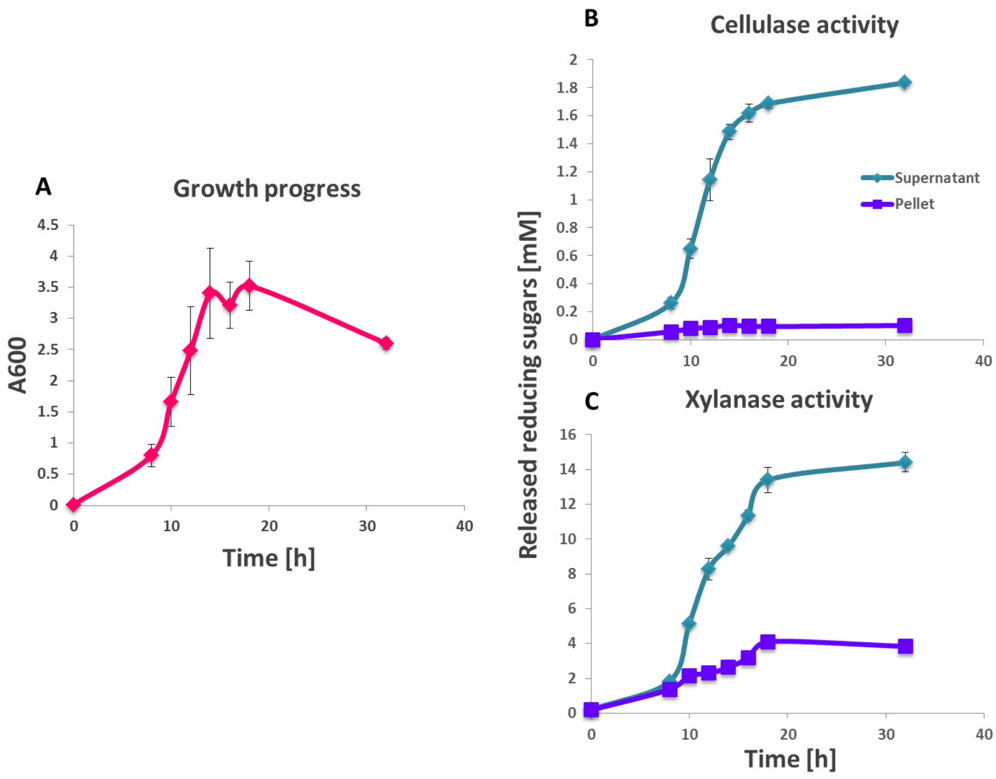

Figure S2. Growth progress and cellulosome production of *C. clariflavum*. Cells were grown on cellobiose-containing medium, and growth progress was assessed by sampling the culture at different time points. (A) Absorbance (A_{600}) at each time point. The graph shows the mean of each time point for three biological repeats. For each sample, the cells were removed from the supernatant fluids, and catalytic activity of both the supernatant and pellet was examined on microcrystalline cellulose (B) and xylan (C). The graphs (Panels B and C) are representative of three biological repeats. Standard deviations are presented.
