# Supplementary material for: Colocalization and Disposition of Cellulosomes in Clostridium clariflavum as Revealed by Correlative Superresolution Imaging
Source: mBio. 2018 Feb 6;9(1):e00012-18. doi: 10.1128/mBio.00012-18 (PMC5801460; doi:10.1128/mBio.00012-18)
Supplement: FIG S3 [file mbo001183712sf3.pdf]

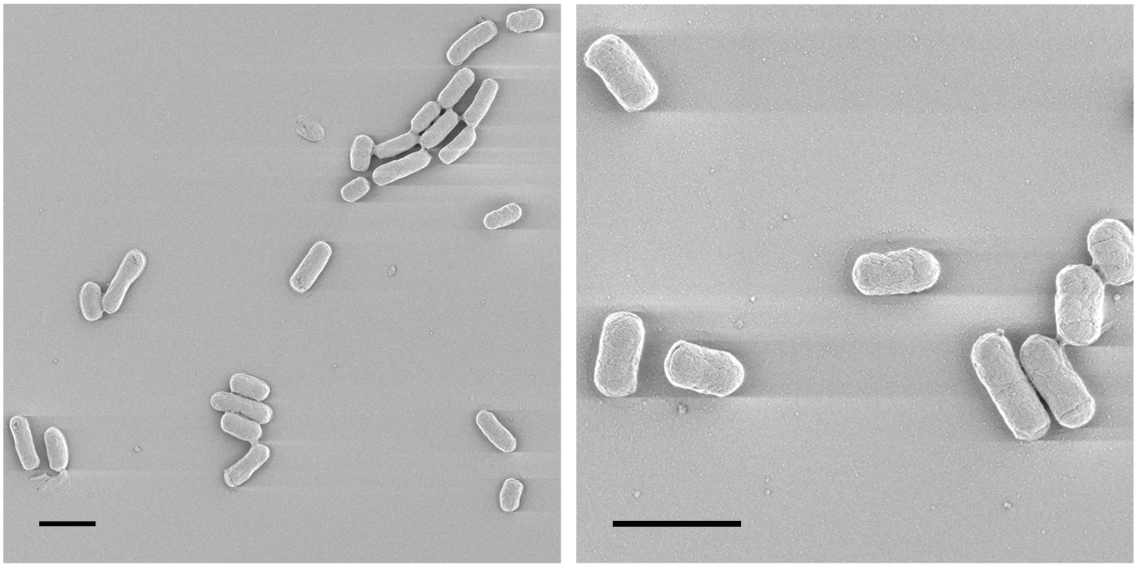

Figure S3. Scanning electron micrograph of *Escherichia coli* cells. *E. coli* cells comprised a negative control, prepared and imaged by SEM using an identical protocol to that of the *C. clariflavum* samples. The cell surface of *E. coli* cell is smooth, thereby contradicting the possibility that the protuberances presented on *C. clariflavum* cells are artifacts of sample preparation. Scale bars: 2 μm.
