# Supplementary material for: Colocalization and Disposition of Cellulosomes in Clostridium clariflavum as Revealed by Correlative Superresolution Imaging
Source: mBio. 2018 Feb 6;9(1):e00012-18. doi: 10.1128/mBio.00012-18 (PMC5801460; doi:10.1128/mBio.00012-18)
Supplement: TEXT S1 [file mbo001183712s1.pdf]

Supplemental Materials and Methods

Cloning, expression and purification of antigens. *C. clariflavum* 19732 genomic DNA was purchased from Leibniz Institute DSMZ-German Collection of Microorganisms and Cell Culture. Gene sequences of GH48, cohesin 1 of ScaA (CohA), cohesin 4 of ScaB (CohB) and cohesin 1 of ScaC (CohC) were cloned into a pET28 cassette (specific primers are listed in Table S1), by Reddymix X 2 (Advanced Biotechnologies Ltd., Epsom, Surrey, UK) using PCR. All protein sequences were designed to contain an N-terminal His-tag, and a tobacco etch virus (TEV)-cleavage site was inserted immediately downstream of the His-tag in CohA, CohB, and CohC. Purification of PCR products was conducted by HiYield gel-PCR fragment extraction kit (Real Biotech Corporation, RBC, Banqiao City, Taiwan). PCR products were inserted into the plasmid by restriction with NcoI and XhoI (FastDigest, Thermo scientific, Fermentas UAB, Vilnius, Lithuania) and ligation. Plasmids were transformed to *E. coli* XL-1 Blue. Plasmid purification was performed by QIAprep Spin Miniprep Kit (QIAGEN GmbH, D-40724 Hillden, Germany). Recombinant plasmids were transformed into *E. coli* BL21 (DE3). Cells were grown in LB (Luria Bertani broth) medium containing 50 μg/mL Kanamycin and 2 mM $CaCl_2$ to $A_{600} \approx 0.8$ for 2.25 h at 37ºC. Induction of protein expression commenced with supplementation of 0.2 mM Isopropyl-1-thio-β-galactoside (IPTG) (Frementas UAB, vilinius, Lithuania). After induction, cultures were transferred to 16°C for 16 h. Cells were centrifuged at 4192.5 X g for 15 min, and resuspended in TBS (Tris-buffered saline, 137 mM NaCl, 2.7 mM KCl, 25 mM Tris–HCl, pH = 7.4), 5 mM Imidazole. Cells were sonicated, sonicates were heated to 50°C for 20 min, and centrifuged again at 25155 x g, 4°C. The purification of the four proteins was accomplished by using Ni-NTA beads in a batch purification system as described earlier (1). CohA, CohB and CohC were cleaved by TEV protease (Chemical Research Support, Weizmann Institute) subsequent to the first purification step in order to remove the His-tags. TEV protease was added to each purified protein solution in 1:20 ratio, and the solution was dialyzed overnight in TBS supplemented with 5 mM $CaCl_2$. A subsequent purification step was conducted to separate the cleaved proteins. The solution was incubated with Ni-NTA beads for 1 h,

4°C. The unbound fraction (containing only the desired cleaved proteins) was collected, and the proteins were subjected to SDS-PAGE for analysis of purity.

CohB and GH48 were used for the production of polyclonal chicken antibodies (Siap Laboratory, Bet Gamliel, Israel), while CohA and CohC were used for production of monoclonal mouse antibodies (Antibody unit, Weizmann Institute).

Fluorescence immunolabeling. Cells, grown for 12 h on cellobiose, were centrifuged at 4700 *g* for 5 min, and washed with PBS (phosphate buffered saline, Biological Industries, Beit Haemek, Israel). Cells, grown on wheat straw or MCC, were first centrifuged at 100 *g* for 5 min to discard the large cellulose or straw residues and then centrifuged again at 4700 *g* for 5 min. Cells were concentrated 40 fold (when grown on cellobiose) or 80 fold (when grown on MCC or WS) and subjected to 1 h of blocking with PBS supplemented with 5% (wt/vol) BSA and 0.05% (vol/vol) Tween 20, rotating at room temperature. Cells were then immunolabeled with pairs of antibodies (one monoclonal and one polyclonal): (i) anti GH48 (1:500) + anti CohA (1:500), (ii) anti-CohA + anti-CohB (1:500), (iii) anti-Coh-B4 + anti-CohC (1:100), and (iv) anti-CohC + anti-GH48, in PBS with 2% (wt/vol) BSA and 0.05% (vol/vol) Tween 20 for 1 h, RT. Cells were washed 6 times with PBS and Tween 20 (washing buffer) and incubated for 1 h with secondary antibodies – anti-mouse conjugated to Alexa Fluor® 568 (1:400) and goat-anti-chicken, conjugated to Alexa Fluor® 647 (1:400) (Jackson ImmunoResearch Laboratories, Inc., West Grove, PA, USA) in PBS with 2% (wt/vol) BSA and 0.05% (vol/vol) Tween 20. Controls were incubated with blocking solution instead of the primary antibody pair and with the secondary antibodies. Cells were washed again 6 times with washing buffer, and post-fixed with 3% paraformaldehyde (EMS, Hatfield, PA) for 15 min, rotating at room temperature. The fixed cells were washed 3 times in PBS and placed on coverslips attached to a Petri dish covered with 0.002% poly-L-lysine (P35G-1.5-14-C, MatTek In Vitro Life Science Laboratories, Bratislava, Slovak Republic). For correlative work, grid-containing coverslips were used (81158, Ibidi, Martinsried, Germany), covered with poly-L-lysine.

Dual-color 3D STORM imaging and analysis. Three-dimensional super resolution imaging was performed using a Vutara SR200 STORM microscope based on single-

molecule localization biplane technology, using a 60x water-immersion objective with 1.2 NA. Dual-color measurements were performed using 647-nm (5 kW/cm^2) and 561-nm lasers (5 kW/cm^2). For each color, 6,000 frames were recorded with acquisition times of 20 ms per frame. Imaging was performed in the presence of imaging buffer (7 μM glucose oxidase (Sigma), 56 nM catalase (Sigma), 2 mM cysteamine (Sigma), 50 mM Tris, 10 mM NaCl, 10% glucose, pH 8). Data were analyzed and visualized by the Vutara SRX software using the following parameters: 50-nm particle size and threshold value (typically set to 5) that was defined as the standard deviation above the mean value of the border pixels in each frame. In addition, de-noise function by Vutara software was used to eliminate scattered points based on nearest neighbor algorithm. Thermal drift was corrected manually if needed. Lateral localization accuracy was estimated by Thompson et al. (2) to be 5.94 ± 3.10 in our measurements.

ELISA assay. The specificity of the produced polyclonal and monoclonal antibodies against the cellulosomal modules was examined by ELISA. 96-well plates (Greiner Bio-One, Belgium) were coated with 1 μg/mL of each antigen (GH48, CohA, CohB, and CohC) and incubated overnight at 4°C. Plates were blocked with TBS containing 2% BSA (wt/vol) and 0.05% (vol/vol) Tween 20 for 1 h, at room temperature, following incubation with the primary antibodies: anti-GH48, anti-CohA, anti-CohB and anti-CohC (1:5000), 1 h, room temperature. Each antigen was incubated with all four antibodies, to examine possible cross-reactivity. Plates were then washed 3 times in TBS supplemented with Tween 20 (TBST) and incubated with anti-mouse and anti-chicken HRP (horseradish peroxidase)-coupled antibodies for 1 h, room temperature. Plates were washed 4 times in TBST, and tetramethylbenzidine (TMB) (Dako, Glostrup Municipality, Denmark) was added to the plates for 3 min. the reaction was stopped by adding 1 N H_2SO_4, and antibody-antigen binding was assessed by measuring absorbance at 450 nm.

***C. clariflavum* growth and cellulosome production.** Cells were grown on cellobiose for 32 h in 1 L fermentors. Samples were collected at different time points, and the cell pellet was separated by centrifugation from the spent growth medium. Optical density was measured for each time point (Figure S2 A). Supernatant fluids at each time point were

washed three times with TBS using Vivaspin® 30 kDa concentrators (Sartorius Stedim Biotech GmbH, Goettingen, Germany) to remove residual cellobiose. Cell pellets were washed in TBS and resuspended in the same buffer to the original volume. Supernatant fractions and cell pellets of each time point were taken at the same volume ratio for activity assays, to check for enzymatic activity. The total volume of each reaction was 200 μL, and contained 50 mM acetate buffer, pH 5.5, 12 mM $CaCl_2$, and 2 mM EDTA. Each reaction contained 28 μL of supernatant fluids or resuspended cell pellet at each time point. Cellulase activity (Figure S2 B) was measured by adding to the reaction 11.25 mg/mL Avicel (Sigma, Rehovot, Israel). The reaction was allowed to proceed for 24 h at 55°C. Xylanase activity was assessed by adding to the reaction beechwood xylan (Sigma Aldrich, Rehovot, Israel) at a final concentration of 1% (w/v), and the reaction was carried out for 1 h, at 55°C (Figure S2 C). The concentration of released reducing sugars was determined by the dinitrosalicylic acid (DNS) method (3).

References

1. Vazana Y, Moraïs S, Barak Y, Lamed R, Bayer E a (2010) Interplay between *Clostridium thermocellum* family 48 and family 9 cellulases in cellulosomal versus noncellulosomal states. *Appl Environ Microbiol* 76(10):3236–43.
2. Thompson RE, Larson DR, Webb WW (2002) Precise nanometer localization analysis for individual fluorescent probes. *Biophys J* 82(5):2775–2783.
3. Miller GL (1959) Use of dinitrosalicylic acid reagent for determination of reducing sugar. *Anal Chem* 31(3):426–428.
