# Supplementary material for: Colocalization and Disposition of Cellulosomes in Clostridium clariflavum as Revealed by Correlative Superresolution Imaging
Source: mBio. 2018 Feb 6;9(1):e00012-18. doi: 10.1128/mBio.00012-18 (PMC5801460; doi:10.1128/mBio.00012-18)
Supplement: TABLE S1 [file mbo001183712st1.pdf]

Table S1. Primers used for cloning of the antigen genes.

Gene	Primer	Sequence
GH48	U-GH48-DOC-NcoI	ttataaCCATGGgccaccatcaccatcaccatgactctg acactttttaaagac
	L-GH48-DOC-XhoI	tatattCTCGAGttaaaaatctttgcttattccg
CohA1	U-CohA1-TEV-NcoI	ttatatCCATGGgccaccatcaccatcaccatgaaaacc tgtattttcagggcgcacaagatgattcgacggc
	L-CohA1-TEV-XhoI	atattaCTCGAGttaagcgtttgttggtgttgc
CohB4	U-CohB4-TEV-NcoI	taatatCCATGGgccaccatcaccatcaccatgaaaacc tgtattttcagggcgcagtaccaacaaccgatag
	L-CohB4-TEV-XhoI	atattaCTCGAGtcaagatattacttcagcat
CohC1	U-CohC1-TEV-NcoI	ttataaCCATGGgccaccatcaccatcaccatgaaaacc tgtattttcagggcgcagggcaattacaaattg
	L-CohC1-TEV-XhoI	atattaCTCGAGtcaatttgagccaaccaatatagc
